# Complete Genome Sequence of NEB 5-alpha, a Derivative of *Escherichia coli* K-12 DH5α

**DOI:** 10.1128/genomeA.01245-16

**Published:** 2016-11-10

**Authors:** Brian P. Anton, Elisabeth A. Raleigh

**Affiliations:** New England Biolabs, Ipswich, Massachusetts, USA

## Abstract

*Escherichia coli* K-12 DH5α is one of the most popular and widely available laboratory strains, but, surprisingly, no complete genome sequence has been publicly available. Here, we report the complete, finished sequence of NEB 5-alpha (DH5α *fhuA2*). It should serve as a useful reference for researchers working with DH5α.

## GENOME ANNOUNCEMENT

DH5α, constructed by Douglas Hanahan, is one of the most commonly used *Escherichia coli* K-12 laboratory strains. It is widely commercially available and has several properties that make it well suited for cloning applications: it can be transformed with high efficiency; yields high-quality plasmid DNA due to the absence of nonspecific endonuclease I (*endA1*) (1); maintains plasmids stably due to low levels of homologous recombination (*recA1*); can be transformed efficiently with unmethylated DNA due to the disruption of endonuclease EcoKI (*hsdR17*); supports blue/white selection due to the alpha-complementable Δ*lacZ58(M15)* allele; and is deficient in periplasmic alkaline phosphatase (PhoA^−^), making it useful for studying membrane proteins expressed as *phoA* fusions (2). Finally, it is the only commercially available M.EcoKI^+^ strain, so DNA passaged through it will efficiently transform classical EcoKI^+^ *E. coli* strains.

Several DH5α markers have been characterized previously: *deoR*, originally thought to be defective and therefore responsible for the high transformability of DH5α (3), is in fact a wild type (4); *luxS* is defective (5); and the *rfbC1* allele is actually a frameshift in *rfbD* (6). Numerous other features can be deduced from the genome sequence of its ancestor, DH1 (7). A whole-genome shotgun assembly (WGA) of DH5α, based on short reads and comprising 89 contigs, revealed additional variants (6). Although the characteristic deletion Δ(*argF-lac*)*169* was originally determined to be 97 kb in length (8), the WGA study (erroneously, we find) inferred a shorter length of 85 kb, with the *cynS-mhpC* region not part of the deletion (6).

We have sequenced the genome of NEB 5-alpha (New England Biolabs), an immediate *fhuA2* derivative of DH5α, using the Pacific Biosciences RSII platform with P6 chemistry. A 10-kb SMRTbell library was prepared from total DNA using the manufacturer’s instructions, size-selected (4 to 50 kb) using the BluePippin instrument (Sage Science), and sequenced on 1 SMRT cell with a 360-min movie, obtaining 314× mean coverage. The genome was assembled using RS_HGAP_Assembly.3 followed by manual refinement and reassembly using RS_BridgeMapper.

The closed and finished genome of NEB 5-alpha is 4,583,637 bp in length. Our assembly shows the deletion at Δ(*argF-lac*)*169* to be 97,240 bp in length, extending from *mmuP* to *mhpD*, which is consistent with the original report (8) and contrary to the WGA study (6). The *cynS-mhpC* region, including the Δ*lacZ58(M15)* allele, is present on the 47,357-bp ϕ80d[Δ*lacZ58*(*M15*)] insertion at *attϕ80* (as in DH10β [9]) but is deleted as part of Δ(*argF-lac*)*169*, which we confirmed by PCR. The *phoA8* allele is a 723-bp internal, in-frame deletion.

In addition to those genes disrupted in DH1, *fhuA*, *crl*, and *phoE* are disrupted in NEB 5-alpha, and *ylbE* contains a frameshift. We confirmed all of the nonsynonymous changes identified previously (6), except for *abgR* and *yicJ*, which appear to be wild type. We identified four additional nonsynonymous mutations: *flgJ* (P254S), *insH20* (W140stop), *msbB* (M33I), and *ppsA* (A50T). We suggest the following genotype: *fhuA2::IS2* Δ(*mmuP-mhpD*)*169* Δ*phoA8 glnX44* ϕ80d[Δ*lacZ58*(*M15*)] *rfbD1 gyrA96 luxS11 recA1 endA1 rph*^WT^ *thiE1 hsdR17*.

### Accession number(s).

This sequence has been deposited at DDBJ/ENA/GenBank under the accession number CP017100 and is also available at New England Biolabs.
